# Synergistic Anticancer Activity of *Annona muricata* Leaf Extract and Cisplatin in 4T1 Triple-Negative Breast Cancer Cells

**DOI:** 10.3390/cells15030213

**Published:** 2026-01-23

**Authors:** Oumayma Kouki, Mohamed Montassar Lasram, Amel Abidi, Jérôme Leprince, Imen Ghzaiel, John J. Mackrill, Taoufik Ghrairi, Gérard Lizard, Olfa Masmoudi-Kouki

**Affiliations:** 1 LR18ES03, Laboratory of Neurophysiology, Cellular Physiopathology and Biomolecules Valorisation, Faculty of Science of Tunis, University Tunis El Manar, Tunis 2092, Tunisia; oumaikouki@gmail.com (O.K.); montassar.lasram@istmt.utm.tn (M.M.L.); amel.abidi@etudiant-fst.utm.tn (A.A.); taoufik.ghrairi@fst.utm.tn (T.G.); 2 Laboratory of Neuroendocrine, Endocrine and Germinal Differentiation and Communication (NorDiC), Inserm UMR 1239, University Rouen Normandie, 76000 Rouen, France; jerome.leprince@univ-rouen.fr; 3 Clermont Auvergne National Polytechnic Institute, INP, French National Centre for Scientific Research, Institut Pascal, University Clermont Auvergne, 63001 Clermont-Ferrand, France; imen.ghzaiel@u-bourgogne.fr; 4 Department of Physiology, University College Cork, Western Gateway Building, Western Road, T12 XF62 Cork, Ireland; j.mackrill@ucc.ie; 5 Laboratoire Interdisciplinaire Carnot de Bourgogne ICB UMR 6303 (Team Prof. N. Millot), French National Center for Scientific Research (CNRS), Inserm, University Bourgogne Europe, 21000 Dijon, France; gerard.lizard@u-bourgogne.fr

**Keywords:** triple-negative breast cancer cell line, *Annona muricata* aqueous leaf extract, cisplatin, synergetic anticancer, apoptosis, autophagy

## Abstract

**Highlights:**

**What are the main findings?**

- *Annona muricata* leaf extract exhibits significant anti-tumor activity in 4T1 breast cancer cells.
- *Annona muricata* leaf induces autophagy-mediated cell death via mTOR downregulation and increased Beclin1 and LC3 expression

**What are the implications of the main finding?**

- Combined treatment with cisplatin shifts cell death from intrinsic apoptosis to autophagy, enhancing anti-cancer efficacy.
- *Annona muricata* leaf extract reduces cisplatin-induced inflammation by inhibiting *TNFα* expression and promoting *IL-10* expression.

**Abstract:**

Breast cancer remains one of the leading causes of cancer-related mortality among women worldwide. Although cisplatin is widely used in chemotherapy, its clinical efficacy is often limited by adverse effects and resistance. Thus, natural bioactive compounds are gaining attention as complementary therapeutic agents. This study aimed to evaluate the anti-tumor effects of *Annona muricata* leaf extract on murine breast cancer 4T1 cells, used alone or in combination with cisplatin. Cisplatin induced intrinsic apoptosis through mitochondrial membrane disruption, up-regulation of the *Bax* gene and inhibition of the PI3K/AKT/mTOR signaling pathway. Cisplatin also promoted hypoxia by HIF1α gene expression, inflammation by *TNFα* and *IL-6* gene expression, and induced cell cycle arrest at the sub-G1 phase by down-regulation of *cyclin D1* and *cyclin E1* genes. *Annona muricata* leaf extract triggered autophagy-mediated 4T1 cell death through mainly *mTOR* down-regulation and increased expression of Beclin1 and *LC3* genes. It also induced cell cycle arrest at sub-G1 and S phases in a concentration- and time-dependent manner. When, combined with cisplatin, *Annona muricata* extract shifts the cell death pathway from intrinsic apoptosis toward autophagy by reduced caspase-3 gene expression and activity and enhanced LC3-I to LC3-II conversion. Moreover, *Annona muricata* extract attenuated cisplatin-induced inflammation by inhibiting *TNFα* and *IL-6* gene expression and reinforced cell cycle arrest through suppression of the *cyclin D1* gene. In conclusion, our results suggest that *Annona muricata* leaf extract exerts significant anti-tumor activity in breast cancer cells and may enhance cisplatin efficacy by shifting the signaling pathway from intrinsic apoptosis toward autophagy, and attenuating inflammation-related effects, supporting its potential use as a complementary therapeutic strategy.

## 1. Introduction

Breast cancer is characterized by uncontrolled cell proliferation of the epithelial tissue either in the ducts, ductal carcinoma or in lobules [1]. Breast cancer is thought to begin with genetic mutations in a single cell which multiplies indefinitely to form a colony, or primary tumor [2]. The primary tumor develops malignant hallmarks, i.e., cell death resistance, proliferative signaling, evasion of the immune system and angiogenesis to become immortal and invasive [3]. Based on immunohistochemical expression of receptors, breast cancer is classified into four major subtypes: luminal breast cancer A and B; breast cancer overexpressing human epidermal growth factor receptor 2 (HER2); and triple negative breast cancer (estrogen and progesterone receptor negative, HER2 negative) [4]. Breast cancer has emerged as the most common cancer in women worldwide, surpassing lung cancer, and is the fourth leading death by cancer. In fact, the Global Cancer Observatory predicts 3 million breast cancer new cases and 1 million annual deaths by 2040 which reveals a serious health burden, especially in low-income countries like Tunisia [5,6]. In this context, the Tunisian Association for Breast Cancer Support (ATAMCS) has reported that one woman in eight is affected by breast cancer, with 1500 new cases diagnosed each year and a mortality rate of 1.3% deaths annually [7].

Multiple therapeutic approaches are employed to treat breast cancer, including surgery, chemotherapy, hormonal therapy in estrogen receptor-positive tumors, and radiotherapy [8]. Since its approval by the Food and Drug Administration, chemotherapy has remained the first-line treatment of cancers [9]. Based on their mechanism of action, chemotherapeutic agents are divided into alkylating agents, antimetabolites, topoisomerase inhibitors and mitotic spindle inhibitors. However, chemotherapeutic efficacy is limited by cancer high heterogeneity, treatment toxicity, and tumor cells resistance [10]. Meanwhile, it has been demonstrated that multi-drug resistance is responsible for approximately 90% of cancer-related death in patients undergoing chemotherapy [11]. This resistance is attributed to several mechanisms, such as increased drug metabolism and elimination, genetic factors, DNA repair mechanisms, or overproduction of inflammatory cytokines such as IL-1β, IL-4, IL-6 and IL-8 [12].

Cisplatin or cis-diamminedichloroplatinum(II) (CDDP), is classified under alkylating agents that are usually administered intravenously to treat various types of cancer, including breast cancer [13]. Upon entering the cell, cisplatin becomes active through mono- or di-hydration, which allows it to bind covalently to DNA, forming adducts via coordination bonds. These adducts cause structural changes in the DNA, thereby inhibiting its replication and transcription [14]. Subsequently, High-Mobility-Group (hMG) proteins recognize the cisplatin–DNA complex, bind to it, and protect it from DNA repair enzymes [15]. Cisplatin forms also RNA bonds disrupting transcriptional processes [16]. Beyond its genomic effects, cisplatin induces oxidative stress by increasing reactive oxygen species (ROS) production and mitochondrial accumulation. ROS interact with Bax (Bcl-2 associated X) to cause mitochondrial DNA damage, and mitochondrial membrane disruption. The mitochondrial membrane disruption leads to release apoptotic factors, activating the intrinsic apoptotic pathway [14]. Despite its potent anti-proliferative effects on tumor cells, the clinical use of cisplatin is limited by significant side effects, including nephrotoxicity, ototoxicity, myelosuppression, gastrointestinal toxicity, hemorrhage, peripheral neuropathy and hearing loss, particularly in younger patients [17,18]. Moreover, the development of drug resistance further reduces its long-term efficacy, highlighting the need for complementary agents that can enhance therapeutic effects. Combination therapies with cisplatin have been explored to overcome resistance and minimize adverse effects. In this context, herbal medicine has emerged as an alternative therapeutic approach. Approximately 80% of the global population uses phytotherapy to treat a range of pathologies, including diabetes, malaria, skin burns, and cancers [19].

*Annona muricata* (AM), is an evergreen plant that typically grows to a height of 5 to 8 m, with a diameter ranging from 15 to 83 cm [20]. It belongs to the Cotyledon group within the Magnoliideae sub-class, the Magnoliales order, the *Annonaceae* family, and the *Annona* genus. AM is native to the Caribbean and Central America but was cultivated in various tropical regions, including Central and South Africa, China, Australia, and parts of Asia, such as India, Thailand, the Philippines, and the Pacific Islands [21]. Depending on the region, AM is known by various names, including Graviola in the Caribbean, Soursop in the Americas, and Corossolier in France [22]. The plant is rich in bioactive molecules, including alkaloids, megastigmanes, phenolics, minerals, and acetogenins. Acetogenins, in particular, are the most abundant compounds in AM extract [19,23]. Over 120 types of acetogenins have been identified in varying quantities, depending on the plant part and harvest period [24,25]. AM is widely utilized in the pharmaceutical, cosmetic, and industrial sectors. The leaves, in particular, exhibit a broad range of beneficial properties, including anti-arthritic, anti-diabetic, hypolipidemic, anti-convulsant, antioxidant, anti-inflammatory, anti-bacterial, and anti-cancer activities [25]. Some studies have highlighted neurotoxic effects of AM and it has been reported that consumption of the seeds extract offers protective effects in Parkinson’s disease [26] whereas toxic effects have been observed in an in vivo model of Parkinson’s disease [27]. At the cellular level, AM has also been reported to induce ATP depletion in striatal neurons by inhibiting mitochondrial complex I.

Given the ability of AM leaf extract to impact cell cycle, cell viability and tumor cell growth, our objective was to associate AM with cisplatin. The goal was to enhance the anti-tumoral effects while minimizing the side effects associated with cisplatin. To this end, an in vitro study was realized on triple negative 4T1 murine breast cancer cells treated with AM alone or in combination with cisplatin. The anti-tumoral activity was studied, and cell death was characterized.

## 2. Materials and Methods

### 2.1. Cell Culture

The 4T1 cell line was purchased from American Tissue Culture Collection (ATCC, Molsheim, France). 4T1 is a murine cell line that imitates fourth stage human triple-negative breast cancer. Cells were cultured in RPMI-1640 medium supplemented with 2 mM L-glutamine, 1% antibiotic cocktail (*v*/*v*, Penicillin/Streptomycin, 10,000 U/10 mg/mL) and 10% fetal bovine serum (FBS, PAN BIOTECH, Aidenbach, Germany). They were maintained at 37 °C, in a humid atmosphere (5% CO_2_) with medium changes twice a week.

### 2.2. Preparation of Annona muricata Leaf Extract

AM leaves were washed with water and air-dried in the shade for over 10 days. Subsequently, the aqueous leaf extract was obtained by using the cold maceration method. Dried leaves were ground into a powder and blended with distilled water. The resulting mixture was incubated for 48 h at +4 °C with constant shaking. Afterward, it was filtered using Whatman paper number 4, then lyophilized. The dry extract was weighed and stored at 4 °C in the dark for further experiments [28].

### 2.3. Cell Viability Assay

Briefly, 5000 4T1 cells were seeded into 96-well plates and allowed to adhere overnight. Cells were treated with cisplatin (Cytopharma, Zaghouan, Tunisia) and AM in serum-free culture medium for 24 and 48 h. Then, they were visualized under an Inverted microscope with transmitted white light and photographed with Flexacam C3 camera (Leica Microsystems, Wetzlar, Germany). Cell viability was determined by measuring Fluorescein diacetate (FDA, Gibco, Invitrogen, Grand Island, NY, USA) fluorescence in 4T1 cells. Cells were incubated with FDA (15 μg/mL) in the dark for 8 min at 37 °C, washed twice with PBS (0.1 M, pH = 7.4) and lysed with lysis buffer (10 mM Tris-HCl, 1% SDS) [29]. Fluorescence intensity was measured with the Synergy LX multi-mode reader (Bio-Tek Instruments, Winooski, VT, USA) at 485/530 nm (excitation/emission).

### 2.4. Combination Index Analysis

The interaction between AM and cisplatin was assessed using the Chou–Talalay method [30]. 4T1 cells were plated into 96-well plate and treated with graded concentrations of AM (50, 500 and 1500 µg/mL), cisplatin (1, 5 and 10 µM) and their combination for 48 h. The treatment interaction was evaluated by calculating the combination index (CI) according Compusyn software, version 1.0 (ComboSyn, Inc., Paramus, NJ, USA). The CI was calculated with the following equation:

$$\mathrm{CI}\;=\;\frac{dAM}{DAM}+\frac{dCDDP}{DCDDP}$$

where DAM and DCDDP represent the concentrations of AM and cisplatin, respectively, in combination that produce the same effect as AM (*dAM*) and cisplatin (*dCDDP*) alone. The compusyn software generates concentration–effect curves, fractional inhibition (Fa)–CI plots, and normalized isobolograms for CI determination. A CI < 1 indicates a synergic effect, CI = 1 an additive effect and CI > 1 an antagonistic effect [30].

### 2.5. Cell Adhesion Assay

Sulforhodamine 101 (SR101) was used exclusively as a red fluorescent dye to label adherent cells for the quantification of cell adhesion. In this context, SR101 was not employed as a cell-type-specific marker, but solely for visualization purposes in the adhesion assay. 4T1 cells, previously seeded into 96-well plates (5000 cells/well, 200 µL) overnight, were incubated with increasing concentrations of cisplatin and AM for 24 and 48 h. Then, they were fixed with 70% ethanol for 20 min at +4 °C, the supernatant was removed and sulforhodamine 101 (1.5 µg/mL, Sigma Aldrich, St. Louis, MO, USA) was added [31]. After 30 min of incubation at 37 °C cells were washed twice with PBS and the fluorescence intensity was measured using Synergy LX multi-mode reader, BioTek (Bio-Tek Instruments, Winooski, VT, USA) at 535/610 nm (excitation/emission).

### 2.6. Characterization of Cell Death Pathways by Annexin V/Propidium Iodide Flow Cytometry

To assess the proportion of apoptotic cells induced by cisplatin, AM alone or in combination, Annexin V-FITC/propidium iodide (PI) staining was performed using the Annexin V/dead cells apoptosis kit (Cohesion Biosciences, London, UK). 4T1 cells seeded into 6-well plates at a density 0.5 × 10^6^ cells/mL were treated with cisplatin (10 µM) with or without AM (50, 500 and 1500 µg/µL) in the FBS-free medium for 24 and 48 h. Then, cells were rinsed with PBS (0.1 M, pH 7.4), trypsinised with Trypsin/EDTA (PAN BIOTECH, Aidenbach, Germany), centrifuged (200× *g*, 5 min), and stained with Annexin V-FITC/PI according to the manufacturer’s instructions. An amount of 10,000 cells/sample were analyzed using the BD LSR Fortessa cytometer (BD Biosciences, Franklin Lakes, NJ, USA) and data were performed using FlowJo V.X.0.7 software. Four cell subpopulations were analyzed: viable cells (AV−/PI−), early apoptotic cells (AV+/PI−), late apoptotic and/or secondary necrotic cells (AV+/PI+), and necrotic/damaged cells (AV−/PI+) [32].

### 2.7. Evaluation of Transmembrane Mitochondrial Potential (ΔΨm)

Transmembrane mitochondrial potential (ΔΨm) was measured using 3,3′- dihexyloxacarbocyanine iodide (DiOC6(3), Gibco, Invitrogen, Grand Island NY, USA) [33]. Fluorochrome accumulation in mitochondria is proportional to ΔΨm. 4T1 cells were seeded into 6-well plates and incubated at 37 °C with fresh serum-free culture medium in the absence or presence cisplatin (10 µM) with or without AM (50, 500 and 1500 µg/µL) for 24 and 48 h. At the end of the incubation, cells were washed with PBS (0.1 M, pH 7.4), trypsinised, centrifuged (200× *g*, 5 min), and stained with DiOC6(3) (40 nM) for 15 min at 37 °C. The green fluorescence was analyzed using a 520/10 nm filter. 10,000 cells of each sample were analyzed using the BD FACSCanto II flow cytometer (BD Biosciences, Franklin Lakes, NJ, USA) and data were processed with FlowJo V.X.0.7 software.

### 2.8. Measurement of Caspases 3/7 Activities

Cells were incubated at 37 °C with fresh serum-free culture medium in the absence or presence cisplatin (10 µM) with or without AM (50, 500 and 1500 µg/µL) for 24 and 48 h. At the end of the experiments, cells were washed twice with PBS and resuspended in TF2-DEVD-FMK solution (AAT Bioquest, Pleasanton, CA, USA) for 1 to 4 h at 37 °C. TF2-DEVD-FMK is a fluorogenic indicator that binds irreversibly to active caspases 3 and 7. Finally, cells were washed with PBS and resuspended in assay buffer to be analyzed using a Digital LSRII flow cytometer with 530/30 nm filter (BD Biosciences, Franklin Lakes, NJ, USA). An amount of 10,000 cells/sample were acquired and data were analyzed using FlowJo V.X.0.7 software.

### 2.9. Western Blotting Analysis

4T1 cells were seeded into T75 flasks at a density of 5 × 10^6^ cell/flask and incubated at 37 °C with fresh serum-free culture medium in the absence or presence cisplatin (10 µM) with or without AM (50 µg/mL) for 24 and 48 h. Supernatants (containing dead cells), as well as viable cells, were collected by Trypsin-EDTA 1×, washed with PBS and lysed in RIPA buffer (Tris-HCl, NaCl, Nonidet NP40, Na deoxycholate, SDS, NaPO4, EDTA) supplemented with Protease inhibitor cocktail, diluted 25×. After 30 min of incubation on ice, cells were centrifuged at 12,000× *g* for 20 min at +4 °C and supernatants (containing total proteins) was measured using bovine serum albumin (BSA, Sigma Aldrich, St. Louis, MO, USA) method [34]. To this end, 70 µg of total protein was separated on a stacking 4% gel then 8 or 14% separating gel (depending on protein weight) and transferred onto nitrocellulose membranes. Non-specific sites were blocked with 5% milk powder dissolved in PBST (PBS, 1% Tween 20, neutral pH) for 1 h. Membranes were incubated with primary antibodies (Table 1) prepared in 5% milk PBST overnight at +4 °C with constant shaking, washed 3 times with PBST and incubated with secondary antibodies. Finally, proteins were revealed using Enhanced chemiluminescence detection kit. Data were obtained with Image Lab software 6.1 (Bio-Rad) and normalized compared with immunostaining for actin.

### 2.10. Quantitative PCR Analysis

4T1 cells were seeded into 6-well plates at a density of 0.5 × 10^6^ cells/well and treated with cisplatin (10 µM) with or without AM (50 µg/mL) for 12 h. This time point was selected to evaluate early molecular responses, as gene expression changes precede detectable cytotoxic effects induced by cisplatin and Annona muricata. Then, cells were washed with PBS (0.1 M, pH 7.4). Total RNA was extracted with Trizol and purified by a NucleoSpin kit (Macherey-Nagel, Bas-Rhin, Hoerd, France). cDNA was synthetized from 1 μg of total RNA with SensiFAST kit (cDNA Synthesis Kit, Bioline GmbH, Luckenwalde, Germany). Quantitative RT-PCR was performed by using Luna Universal One-Step RT-qPCR Kit (New England Biolabs, Ipswich, England) forward and reverse primers (Table 2), according to the following steps: 55 °C for 10 min, 95 °C for 70 s, 60 °C for 30 s, then 95 °C for 5 min. cDNA was quantified with SYBER Green and calculated using the comparative cycle threshold (Ct) method using GAPDH as an internal control.

### 2.11. Cell Clonogenic Survival Assay

The cell clonogenic survival assay is a basic method used to define cell chemo-sensibility [35]. It enables us to evaluate the occurrence risk, which is the cell capacity to retain reproductive integrity after treatment [36]. Briefly, 1000 4T1-survived cells treated with cisplatin, AM alone and in combination for 24 and 48 h were counted on Malassez chamber using trypan blue (0.05%/0.02%, *v*/*v*), and then cultivated in Petri dish with 100 mm diameter. The medium was changed twice a week. After 6 days, colonies were stained with crystal violet (1%, *w*/*v*) for 5 min, and washed with PBS to eliminate excess crystal violet.

### 2.12. Cell Cycle Analysis by Flow Cytometry

4T1 cells were seeded in 6-well plates at a density of 0.5 × 10^6^ cells/well, then incubated at 37 °C with fresh serum-free culture medium in the absence or presence cisplatin (10 µM) with or without AM (50, 500 and 1500 µg/µL) for 24 and 48 h. At the end of the incubation, cells were washed with PBS (0.1 M, pH 7.4), trypsinised and centrifuged (200× *g*, 5 min). The cell pellets were resuspended in 70% ethanol and stored at −20 °C for 12 h. Finally, cells were stained with the combination PI-RNAse A (80 µg/mL, 200 µg/mL, Gibco, Invitrogen, Grand Island NY, USA) for 1 h [37]. The red fluorescence was detected using a 630 nm band-pass filter and 10,000 cells/sample were analyzed using Digital LSRII flow cytometer (BD Biosciences, Franklin Lakes, NJ, USA). Data analysis was performed using FlowJo V.X.0.7 software.

### 2.13. Statistical Analysis

Statistical analyses were carried out using GraphPad Prism software, version 8.0.1. All data obtained were expressed as means ± SD and analyzed using one-way ANOVA test. A *p*-value of 0.05 or less was considered statistically significant. The inhibitory concentration 50 (IC_50_) was calculated using the four-parameter logistic equation GraphPad Prism.

## 3. Results

### 3.1. Annona muricata and Cisplatin Inhibit 4T1 Cell Viability

**Figure 1:**
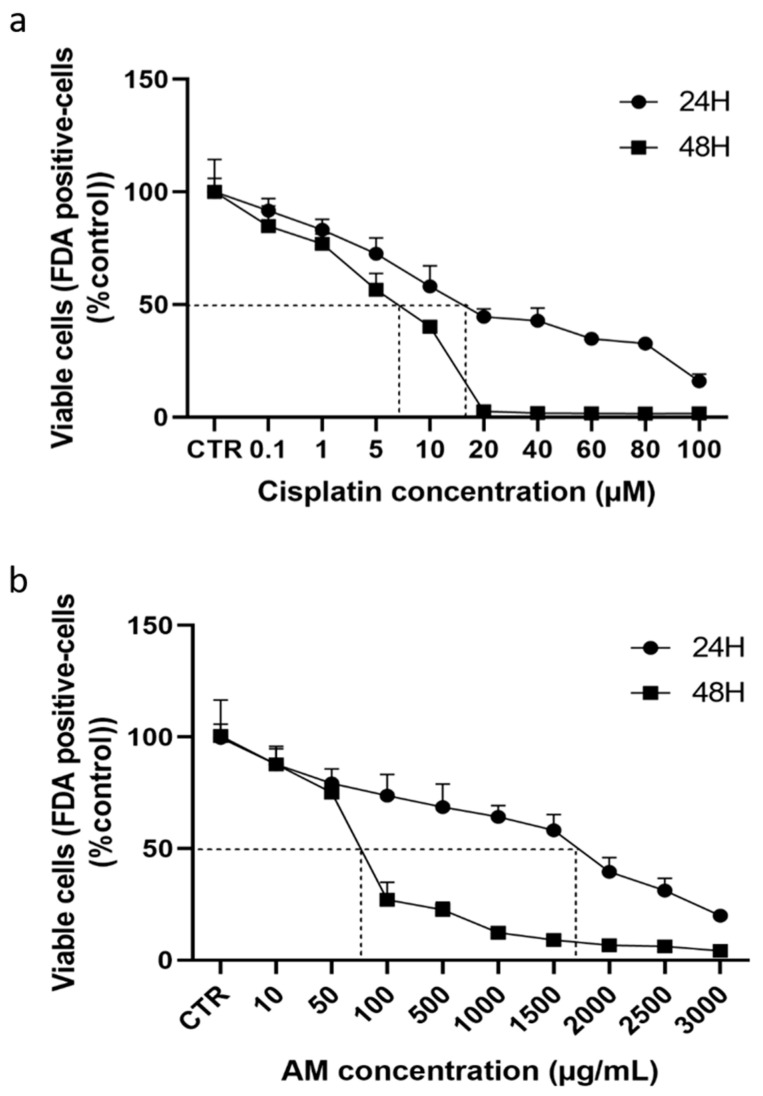
Effect of cisplatin and *Annona muricata* on 4T1 cell viability. 4T1 cells were treated with increasing concentrations of cisplatin (0.1–100 µM), (**a**) or *Annona muricata* leaf extract (10–3000 µg/mL), (**b**) for 24 and 48 h. Cell viability was quantified by FDA fluorescence intensity and expressed as percentage of the control (untreated cells). Data represent mean ± SEM of eight wells from three independent experiments. Statistical analysis was performed using one-way ANOVA followed by Bonferroni’s test. The dashed line corresponds to the 50% effect value of cisplatin and Annona muricata leaf extract.

As previously reported, treatment of 4T1 cells with increasing concentrations of cisplatin for 24 and 48 h resulted in a concentration- and time-dependent decrease in cell viability, with IC_50_ values of 14.70 ± 1.57 µM and 7.18 ± 0.83 µM, respectively. In the present study, we demonstrate that treatment of 4T1 cells with graded dilutions of AM aqueous leaf extract (ranging 10 to 3000 µg/mL) also led to a concentration-dependent reduction in cell viability, with IC_50_ values of 1639.93 ± 84.93 µg/mL after 24 h and 72.53 ± 3.54 µg/mL after 48 h of incubation (Figure 1).

**Figure 2:**
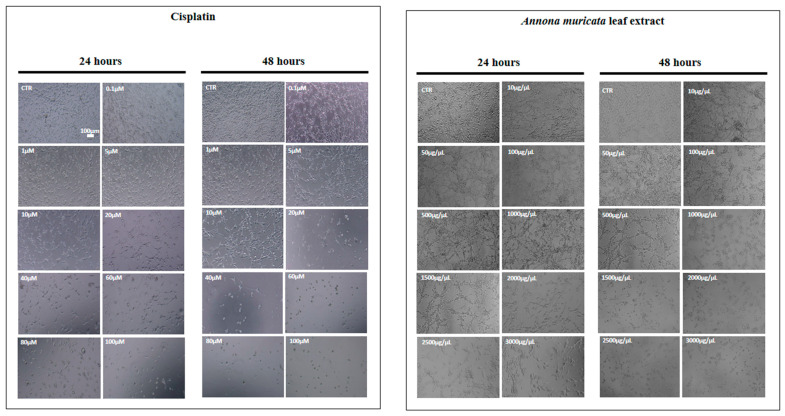
Phase-contrast micrographs showing the effect of cisplatin and *Annona muricata* on 4T1 cell morphology. Morphological changes of 4T1 cells were analyzed by phase-contrast microscopy after treatment with cisplatin (0.1–100 µM) or *Annona muricata* leaf extract (10–3000 µg/mL) for 24 and 48 h. CTR: untreated cells. Images were captured at ×10 magnification.

Phase-contrast microscopic analysis revealed a marked reduction in cell density following treatment with both cisplatin and *AM* extract, evident by the presence of large empty areas within the culture monolayer at both time periods. Morphological changes were characteristic of cytotoxic effects, including cell shrinkage, retraction of cytoplasmic processes, and loss of the typical fusiform shape. Treated cells appeared rounded, darker, and detached from the substrate (Figure 2).

### 3.2. Annona muricata Enhances Cisplatin’s Effect on 41T Cells

To evaluate the effect of AM on the chemosensitivity of 4T1 cells to cisplatin, the interaction between both treatments was assessed using compusyn software. 4T1 cells were incubated with different concentrations of cisplatin, AM alone and in combination, for 48 h and the cell viability was determined by FDA assay. Then, the CI was calculated by the Chou–Talalay method and interpreted as following CI < 1 synergic, CI = 1 additive and CI > 1 antagonistic effects. The AM–cisplatin combination demonstrated a synergic interaction after 48 h (CI = 0.835 < 1) (Table 3).

### 3.3. Annona muricata and Cisplatin Inhibit Cell Adhesion Before Inducing Cell Death

**Figure 3:**
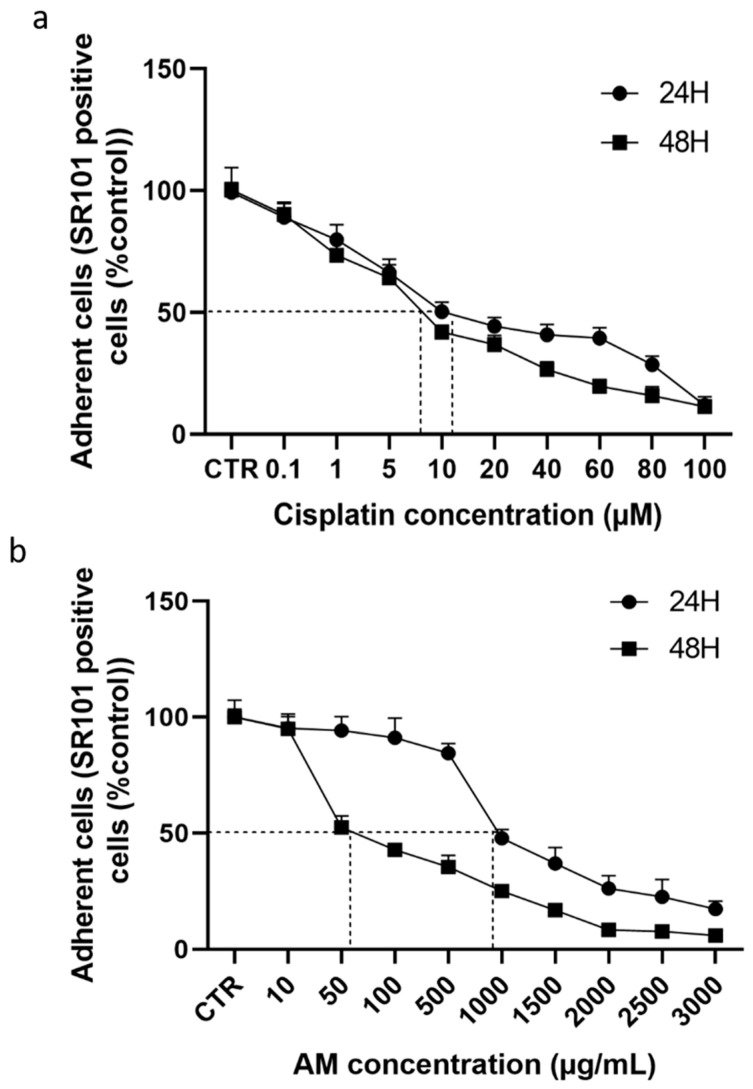
Effect of cisplatin and *Annona muricata* on 4T1 cell adhesion. 4T1 cells were treated with graded concentrations of cisplatin (0.1–100 µM) (**a**) or *Annona muricata* leaf extract (10–3000 µg/mL) (**b**) for 24 and 48 h. Data are expressed as mean percentages ± SEM of *n* = 8 from three independent experiments, with the CTR group (untreated cells) considered 100% adhesion. CTR: untreated cells; AM: *Annona muricata* leaf extract. The dashed line corresponds to the 50% effect value of cisplatin and *Annona muricata* leaf extract.

Sulforhodamine 101 (SR101) is an anionic dye that binds to membrane proteins involved in cell adhesion through electrostatic interactions. As shown in Figure 3, both AM extract and cisplatin impair cell adhesion in a concentration-dependent manner. The IC_50_ values for AM were 952.00 ± 71.60 µg/mL after 24 h and 57.66 ± 6.45 µg/mL after 48 h, while those for cisplatin were 13.19 ± 1.25 µM and 7.35 ± 0.63 µM, respectively. These results highlight a marked increase in the anti-adhesion action of AM after 48 h of treatment (more than 16-fold). IC_50_ values for cell adhesion were consistently lower than those for cell viability, suggesting that loss of adhesion precedes cell death in 4T1 cells.

### 3.4. Characterization of Cell Death Pathway Induced by Annona muricata, Cisplatin Alone and in Combination on 4T1 Cell Line

**Figure 4:**
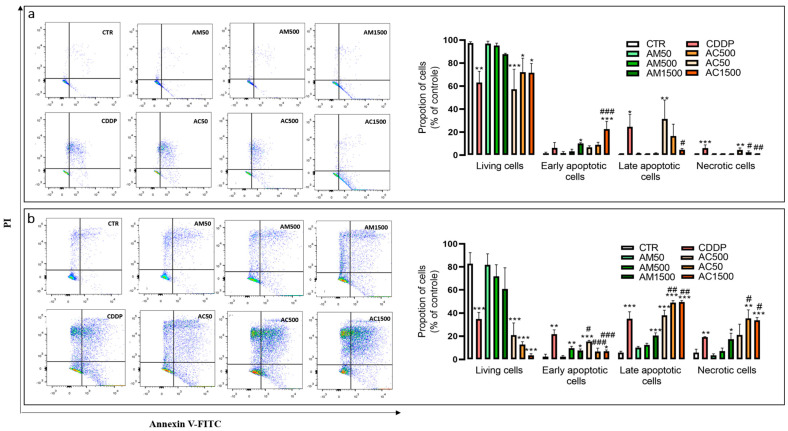
Effect of cisplatin and *Annona muricata* on apoptosis of 4T1 cells. Flow cytometric analysis of 4T1 cells double-stained with Annexin V-FITC (AV) and propidium iodide (PI). 4T1 cells were treated with cisplatin (10 µM) and/or *Annona muricata* leaf extract (50, 500 and 1500 µg/mL) for 24 h (**a**) and 48 h (**b**). Viable cells (AV−/PI−) are located in the lower left quadrant, early apoptotic cells (AV+/PI−) in the lower right, late apoptotic and/or secondary necrotic cells (AV+/PI+) in the upper right, and necrotic/damaged cells (AV−/PI+) in the upper left. The color scale in the flow cytometry quadrants represents cell population density, ranging from low to high, from blue to red. Data are expressed as mean percentages ± SEM from three independent experiments performed in triplicate. Statistical analysis was performed using one-way ANOVA followed by Bonferroni’s test (* *p* < 0.05, ** *p* < 0.01, *** *p* < 0.001 vs. CTR and # *p* < 0.05, ## *p* < 0.01, ### *p* < 0.001 vs. CDDP). CTR: untreated cells; CDDP: Cisplatin (10 µM); AM50, AM500, AM1500: *Annona muricata* (50, 500 and 1500 µg/mL); AC50, AC500, AC1500: cisplatin (10 µM) combined with *Annona muricata* (50, 500 and 1500 µg/mL). The black lines in the flow cytometry quadrants correspond to the threshold (limit) for fluorescence intensity detection.

To further discriminate apoptotic from necrotic cell death, 4T1 cells were double-stained with annexin V (A V) and PI, and analyzed by flow cytometry to quantify viable (A V^−^/PI^−^), early apoptotic (A V^+^/PI^−^), late apoptotic (A V^+^/PI^+^), and necrotic cells (A V^−^/PI^+^). As illustrated in Figure 4, treatment with cisplatin alone resulted in the following distribution: 6.09 ± 2.12% necrotic cells, 24.56 ± 7.88% late apoptotic cells, 6.31 ± 3.56% early apoptotic cells and 63.13 ± 7.33% viable cells after 24 h; 19.25 ± 0.21% necrotic cells, 35.09 ± 4.59% late apoptotic cells, 21.64 ± 2.87% early apoptotic cells and 34.83 ± 3.76% viable cells after 48 h. Notably, combined treatment with cisplatin and AM extract at a concentration of 1500 µg/mL led to a marked increase in early apoptotic cells compared to cisplatin alone (22.64 ± 5.10% vs. 6.31 ± 3.56%), indicating potentiation of apoptosis. However, co-treatment with AM and cisplatin induces an increase in late apoptotic cells compared to cisplatin alone (49.50 ± 0.93% vs. 35.09 ± 4.59%) and a corresponding rise in necrotic cells (33.68 ± 1.59% vs. 19.25 ± 0.21%). AM treatment alone also induced a concentration-dependent increase in cell death by early apoptosis after 24 h; late apoptosis and necrosis after 48 h when compared to untreated controls. Moreover, AM combined with cisplatin significantly compromised membrane integrity, as evidenced by the increased proportions of A V^+^/PI^+^ and A V^−^/PI^+^ cells, suggesting enhanced apoptotic and necrotic damages. These findings support the pro-apoptotic potential of *AM* and its capacity to sensitize 4T1 cells to cisplatin-induced cytotoxicity.

**Figure 5:**
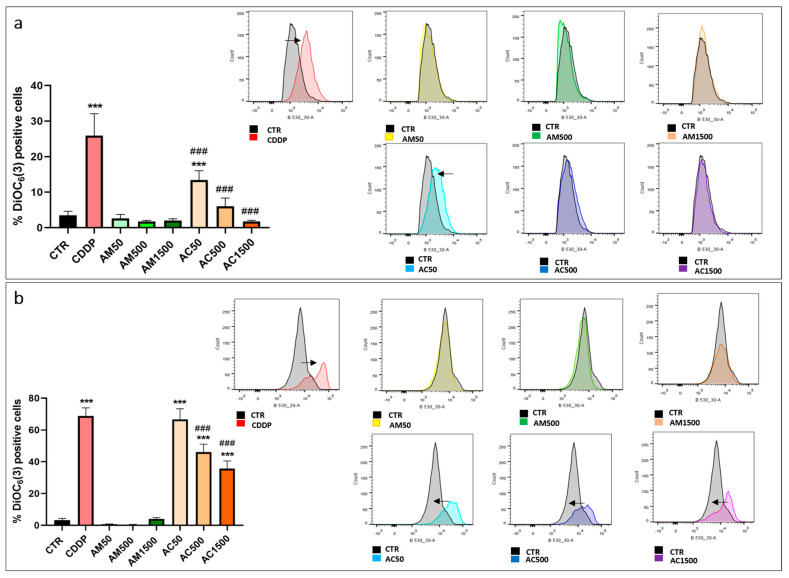
Measurement of mitochondrial transmembrane potential (ΔΨm) in 4T1 cells by flow cytometry with DiOC6(3) staining. 4T1 cells were treated with cisplatin (10 µM) and/or *Annona muricata* leaf extract (50, 500 and 1500 µg/mL) for 24 h (**a**) and 48 h (**b**). Results were analyzed relative to the CTR group, representing normal ΔΨm. A rightward shift indicates hyperpolarization, while a leftward shift indicates depolarization of the mitochondrial membrane. The arrow indicates the shift of the curve in treated cells compared to untreated cells. Data represent mean ± SEM from three independent experiments performed in triplicate. Statistical analysis was performed using one-way ANOVA followed by Bonferroni’s test (*** *p* < 0.001 vs. CTR and ### *p* < 0.001 vs. CDDP). CTR: untreated cells; CDDP: Cisplatin (10 µM); AM50, AM500, AM1500: *Annona muricata* (50, 500 and 1500 µg/mL); AC50, AC500, AC1500: cisplatin (10 µM) combined with Annona muricata (50, 500 and 1500 µg/mL).

To better understand the mechanism(s) by which AM enhances cisplatin-induced apoptosis, we first have investigated mitochondrial integrity by assessing ΔΨm using the fluorescent probe DiOC6(3). As shown in Figure 5, treatment with cisplatin (10 µM) induced a marked mitochondrial hyperpolarization, revealed by a significant accumulation of the probe within the cytoplasm, indicating severe mitochondrial dysfunction. In contrast, AM treatment alone did not significantly alter transmembrane mitochondrial potential. Interestingly, co-treatment with AM was able to attenuate the cisplatin-induced mitochondrial hyperpolarization, as demonstrated by a progressive restoration of DiOC6(3) positive cell populations, suggesting that AM can partially reverse or prevent cisplatin-induced mitochondrial damage.

**Figure 6:**
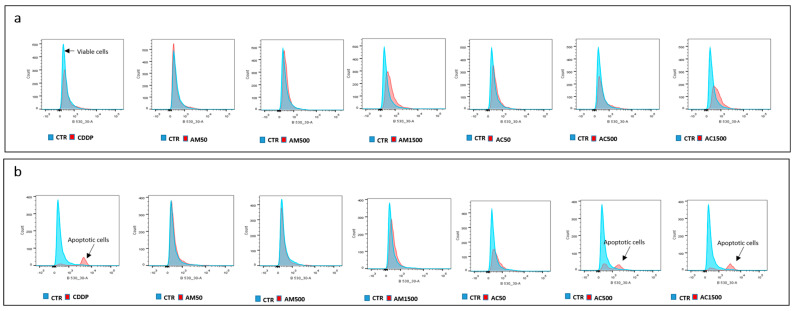
Effect of cisplatin and *Annona muricata* on caspase-3/7 activity in 4T1 cells. 4T1 cells were treated with cisplatin (10 µM) alone or in combination with *Annona muricata* leaf extract (50, 500 and 1500 µg/mL) for 24 h (**a**) and 48 h (**b**). Caspase-3/7 activity was measured relative to the CTR group (untreated viable cells). The appearance of an additional curve indicates activation of caspase-3/7-dependent apoptosis. Data represent mean ± SEM from three independent experiments performed in triplicate. CTR: untreated cells; CDDP: cisplatin (10 µM); AM50, AM500, AM1500: *Annona muricata* (50, 500 and 1500 µg/mL); AC50, AC500, AC1500: cisplatin (10 µM) combined with *Annona muricata* (50, 500 and 1500 µg/mL).

We next investigated the effects of cisplatin and AM alone and in combination on caspase-3/7 activity in 4T1 cells. Treatment with cisplatin alone resulted an increase in caspase-3/7 activity after 48 h of incubation, indicating activation of the apoptotic cascade. In contrast, AM alone induced cell death via a caspase-3/7-independent pathway, suggesting the involvement of alternative mechanisms such as caspase-independent apoptosis. Interestingly, co-treatment with cisplatin and AM did not significantly affect caspase-3/7 activity at 24 h. However, after 48 h the response was concentration-dependent. At a low concentration (50 µg/mL), AM inhibited cisplatin-induced caspase activation, suggesting a transient protective effect and/or interactions with early apoptotic signaling. Conversely, higher concentrations of AM (500 and 1500 µg/mL) enhanced caspase-3/7 activity, indicating a synergistic pro-apoptotic effect with cisplatin at elevated concentrations (Figure 6). These results suggest that AM modulates apoptosis in a concentration- and time-dependent manner, potentially shifting the balance between caspase-dependent and caspase-independent pathways.

### 3.5. Identification of Intracellular Pathway Involved in the Anti-Tumor Effect of Cisplatin, Annona muricata and Their Interaction

**Figure 7:**
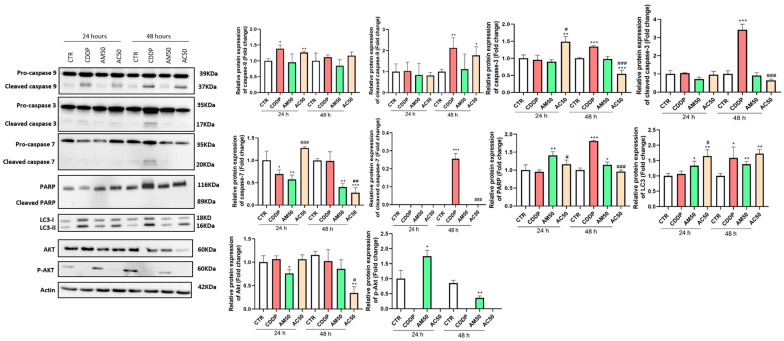
Effect of cisplatin and *Annona muricata* on protein expression in 4T1 cells. 4T1 cells were treated with cisplatin (10 µM) and/or *Annona muricata* leaf extract (50 µg/mL) for 24 and 48 h. Proteins were separated by SDS-PAGE, transferred to nitrocellulose membranes, and probed with specific antibodies. Actin served as the internal loading control. Data are presented as mean ± SEM from three independent experiments. Statistical analysis was performed using one-way ANOVA followed by Bonferroni’s test (* *p* < 0.05, ** *p* < 0.01, *** *p* < 0.001 vs. CTR and # *p* < 0.05, ## *p* < 0.01, ### *p* < 0.001 vs. CDDP). CTR: untreated cells; CDDP: Cisplatin (10 µM); AM50: *Annona muricata* (50 µg/mL); AC50: cisplatin (10 µM) combined with *Annona muricata* (50 µg/mL).

To elucidate the mechanisms by which AM induces 4T1 cell death, alone or in combination with cisplatin, we analyzed the expression of key proteins involved in cell death pathways by Western blotting. As shown in Figure 7, cisplatin induces cell death predominantly via the intrinsic (mitochondrial) caspase-dependent apoptotic pathway. Specifically, it activates pro-caspase-9 after 24 and 48 h of treatment, with cleavage into active caspase-9 only observed at 48 h. This active form subsequently cleaves and activates caspase-3. Furthermore, cisplatin stimulates PARP expression without inducing its cleavage, even after 48 h of exposure. In contrast, AM appears to promote cell death through a distinct mechanism. AM induces autophagy, as evidenced by the conversion of LC3-I to LC3-II, and concurrently inhibits caspase-9 activation at 48 h and caspase-7 activity at both 24 and 48 h. Additionally, AM activates PARP without promoting its cleavage at either time point, suggesting a caspase-independent cell death pathway. Interestingly, AM also interferes with cisplatin-induced caspase activation, while potentiating cell death via autophagy. After 24 h of co-treatment with AM and cisplatin, PARP activation without cleavage is detected, consistent with the shift towards a non-apoptotic mechanism. Finally, cisplatin alone and in combination with AM suppresses Akt phosphorylation, a key effector in the PI3K/Akt/mTOR signaling pathway. In contrast, phosphorylation of Akt remains preserved in cells treated with AM alone, suggesting a potential cytoprotective or regulatory role of AM on this pathway.

**Figure 8:**
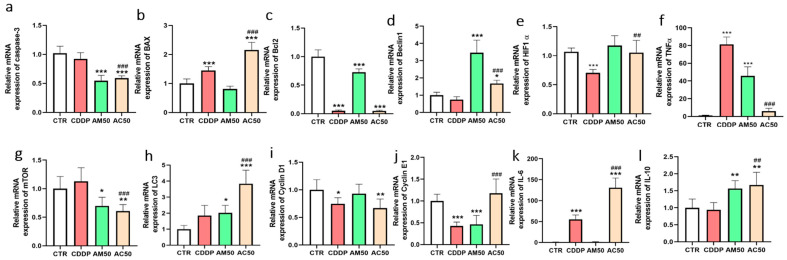
Effect of cisplatin and *Annona muricata* on the expression of pro- and anti-apoptotic, autophagic and inflammatory genes. 4T1 cells are incubated the presence or absence of cisplatin (10 µM), *Annona muricata* (50 µg/µL) and cisplatin (10 µM) plus *Annona muricata* for 12 h. mRNA levels of *caspase-3* (**a**), *Bax* (**b**), *Bcl-2* (**c**), *Beclin1* (**d**), *mTOR* (**e**), *LC3* (**f**), *cyclin D1* (**g**), *Cyclin E1* (**h**), *HIF1 α* (**i**), *TNFα* (**j**), *IL-6* (**k**), *IL-10* (**l**), and were quantified by RT-PCR and normalized to *GAPDH* expression used as an internal control. Data are expressed as means of percentages (±SEM) of three independent experiments performed in triplicate, One-way ANOVA followed by a Bonferroni test were applied. * *p* < 0.05, ** *p* < 0.01, *** *p* < 0.001 compared to CTR group and ## *p* < 0.01, ### *p* < 0.001 vs. CDDP. CTR for untreated cells; CDDP: Cisplatin; AM50 for *Annona muricata* leaf extract and AC50 for cisplatin combined with *Annona muricata* (50 µg/mL).

To further investigate the molecular mechanisms underlying AM-induced cell death, we analyzed the expression of key apoptotic, autophagic, and inflammatory genes by quantitative RT-qPCR. As shown in Figure 8, our results demonstrate that cisplatin had no significant effect on *caspase-3* gene expression compared to the untreated control group. In contrast, AM alone, as well as in combination with cisplatin, significantly downregulated *caspase-3* expression after 12 h of treatment, suggesting a caspase-independent mode of action. Regarding the regulation of the Bcl-2 family, cisplatin, both alone or combined with AM, induced a marked upregulation of the pro-apoptotic gene *Bax*, while concurrently downregulating the anti-apoptotic gene *Bcl-2*, consistent with the activation of the intrinsic apoptotic pathway. Interestingly, AM alone did not alter *Bax* expression, but led to a moderate decrease in *Bcl-2* expression, indicating a partial shift in the pro-/anti-apoptotic balance. In terms of autophagy-related markers, *Beclin-1* expression was significantly induced by AM alone and, to a lesser extent, in combination with cisplatin. Moreover, expression of the mammalian target of rapamycin (*mTOR*), a known inhibitor of autophagy, was repressed in cells treated with AM, either alone or in combination with cisplatin. As a consequence, the expression of *LC3*, a gene coding for the autophagy-related protein LC3-I/II, was also reduced, in agreement with the post-transcriptional accumulation of LC3-II observed previously. In accordance with hypoxic and inflammatory responses, only cisplatin significantly upregulated *HIF-1α*, indicating the induction of cellular hypoxia in 4T1 cells. Additionally, cisplatin promoted an inflammatory response by increasing the expression of pro-inflammatory cytokine genes, *TNF-α* and *IL-6*. Conversely, AM alone led to a milder increase in *TNF-α* and *IL-6* expression but significantly upregulated the anti-inflammatory *IL-10* cytokine. Notably, the cisplatin-AM combination attenuated the inflammatory response, as reflected by the downregulation of *TNF-α* and *IL-6*, alongside an increase in *IL-10* expression, suggesting a synergistic anti-inflammatory effect.

### 3.6. Synergistic Inhibitory Effect of Annona muricata in Combination with Cisplatin on 4T1 Clonogenicity and Cell Cycle Progression

**Figure 9:**
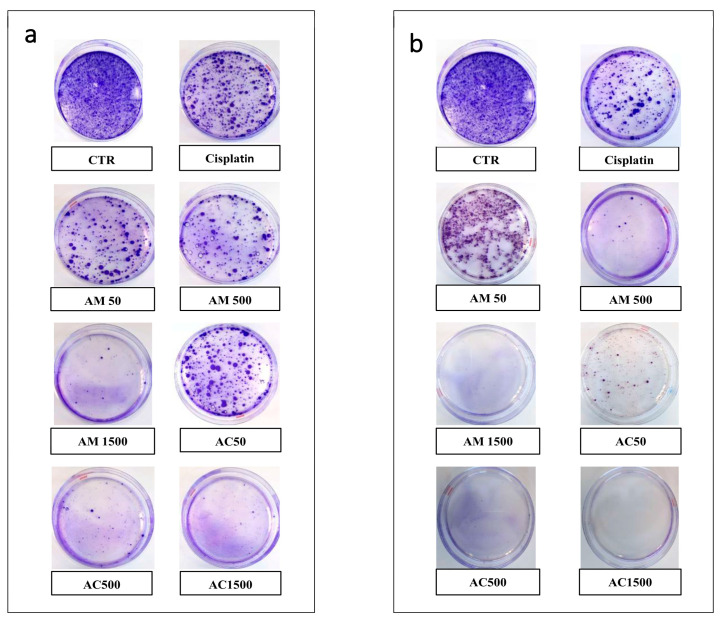
Effect of cisplatin and *Annona muricata* on clonogenic survival of 4T1 cells. 4T1 cells were exposed to cisplatin (10 µM) and/or *Annona muricata* leaf extract (50, 500 and 1500 µg/mL) for 24 h (**a**) and 48 h (**b**). After treatment, 1000 viable cells were reseeded and allowed to grow for 6 days. Colonies were stained with crystal violet. CTR: untreated cells; Cis: cisplatin (10 µM); AM50, AM500, AM1500: *Annona muricata* (50, 500 and 1500 µg/mL); AC50, AC500, AC1500: combination of cisplatin with *Annona muricata* (50, 500 and 1500 µg/mL).

Clonogenic assays were performed to evaluate the long-term proliferative potential of 4T1 cells following treatment. After 24 and 48 h of exposure to cisplatin (10 µM), a substantial number of surviving 4T1 cells retained their proliferative integrity, as evidenced by their ability to form colonies, indicating incomplete elimination of tumorigenic potential (Figure 9). Furthermore, the clonogenic capacity decreased progressively with increasing concentrations of AM after 24 h of incubation and more significantly after 48 h. Notably, the addition of AM markedly reduced the recurrence risk of cisplatin-treated cells. Interestingly, co-treatment with cisplatin and AM at 500 and 1500 µg/mL for 48 h resulted in a complete inhibition of colony formation, with no detectable colonies, highlighting a synergistic effect that significantly compromised the survival and proliferation of 4T1 cells.

**Figure 10:**
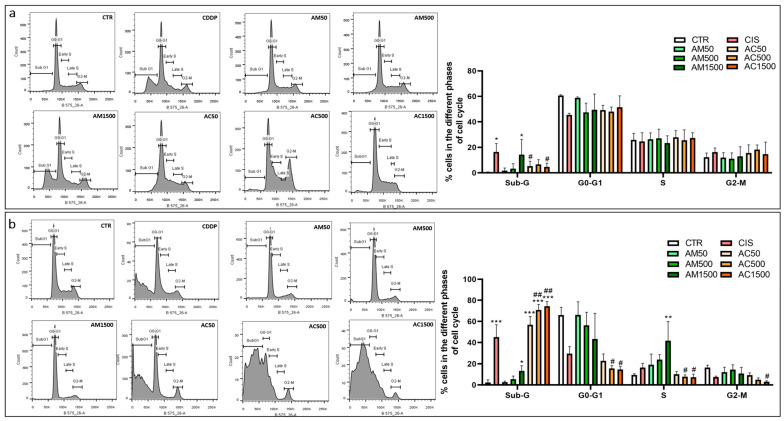
Effect of cisplatin and *Annona muricata* on cell cycle progression of 4T1 cells. Distribution of 4T1 cells in different phases of the cell cycle after 24 h (**a**) and 48 h (**b**) of treatment with cisplatin (10 µM), *Annona muricata* leaf extract (50, 500 and 1500 µg/mL), alone or in combination. Data represent mean percentages ± SEM of three independent experiments performed in triplicate. Statistical analysis was performed using one-way ANOVA followed by Bonferroni’s test. * *p* < 0.05, ** *p* < 0.01, *** *p* < 0.001 vs. CTR and # *p* < 0.05, ## *p* < 0.01 vs. CDDP. CTR: untreated cells; CDDP: Cisplatin (10 µM); AM50, AM500, AM1500: *Annona muricata* (50, 500 and 1500 µg/mL); AC50, AC500, AC1500: cisplatin (10 µM) combined with *Annona muricata* (50, 500 and 1500 µg/mL).

Flow cytometry analysis using PI staining was performed to evaluate the distribution of 4T1 cells across different phases of the cell cycle (Sub-G1, G0/G1, S, G2/M) based on DNA content. As shown in Figure 9, cisplatin induced a marked accumulation of cells in the Sub-G1 phase at both 24 and 48 h, indicating apoptotic DNA fragmentation. The effect of AM on the cell cycle varied depending on the duration of exposure. At a concentration of 1500 µg/mL, AM arrested 4T1 cells at Sub-G1 after 24 h, while after 48 h, a significant accumulation was observed in the Sub-G1 and S phases. Interestingly, co-treatment with AM and cisplatin altered the cell cycle profile of 4T1 cells. After 24 h, AM appeared to modulate the cisplatin-induced Sub-G1 arrest, while after 48 h, it enhanced the cell cycle arrest in a dose-dependent manner, thereby potentiating cytostatic effect of cisplatin. These findings were corroborated by RT-qPCR analysis of cell cycle regulatory genes. After 12 h of incubation, both cisplatin alone or in combination with AM significantly downregulated *cyclin D1* gene expression. In contrast, AM alone had no significant effect on *cyclin D1* mRNA levels compared to the control group. Regarding *cyclin E1*, both cisplatin and AM treatments independently reduced its expression, yet their combination normalized *cyclin E1* mRNA levels, suggesting a possible compensatory or regulatory interaction (Figure 10).

## 4. Discussion

Breast cancer remains a major public health concern in women due to its high incidence, heterogeneity, and frequent resistance to conventional therapies. Among standard treatments, platinum-based agents, such as cisplatin, are commonly used, with nearly 70% of patients receiving this drug [13,38]. In parallel, growing interest in plant-based therapies has led to the exploration of medicinal plants as complementary or alternative cancer treatments. AM, known for its anticancer properties, is one such plant endorsed by WHO guidelines for safe medicinal plant use [39]. The present work studies the combined use of cisplatin and aqueous leaf extract of AM, with the aim of potentiating their anti-tumor efficacy while minimizing cisplatin-induced toxicity.

Our findings demonstrate that cisplatin significantly inhibited the viability and adhesion of 4T1 breast cancer cells in a concentration- and time-dependent manner after 24 and 48 h of exposure, compared to untreated controls. Notably, the lower IC_50_ value for adhesion compared to proliferation suggests that cisplatin disrupts cell–matrix interactions before inducing cell death, which may impair metastatic potential early in treatment. Cisplatin induced cell death primarily through the intrinsic mitochondrial apoptotic pathway, as evidenced by disruption of ΔΨm, leading to the mitochondrial release of cytochrome c, Apoptosis Inducing Factor (AIF), Smac/DIABLO, and other apoptogenic factors into the cytosol. These mediators promote the formation of the apoptosome complex, which in turn activates caspase-9 within 24 h following cisplatin exposure. Caspase-9 activation is followed by the cleavage of effector caspases-3 and -7, key executioners of apoptosis [40]. Despite caspase-3 protein activation, its gene expression was not upregulated, suggesting post-translational regulation or activation from pre-existing protein pools. Caspase-3 activation mediates hallmark features of apoptosis, including DNA fragmentation, cell shrinkage, and membrane inversion, leading to phosphatidylserine externalization and subsequent recognition and clearance by macrophages and parenchymal cells [41]. Moreover, cisplatin upregulated *Bax* gene expression and downregulated *Bcl-2* gene expression, promoting mitochondrial outer membrane permeabilization [42]. Moreover, Bax was also implicated in the activation of autophagy, as shown by the early increased *LC3* mRNA levels at 12 h, and the conversion of LC3-I to LC3-II, a key autophagy marker, at 24 and 48 h [43]. Concurrently, *mTOR*, a negative regulator of autophagy, was suppressed. Cisplatin also inhibited Akt1 phosphorylation at both 24 and 48 h, accompanied by a significant downregulation of transcription factors such as *HIF-1α*. HIF-1α regulates multiple cellular processes including angiogenesis, proliferation and metabolic adaptation under hypoxic conditions [44].

Flow cytometric cell cycle analysis revealed that cisplatin induced a prominent arrest at the sub-G1 phase after 24 and 48 h of treatment, consistent with apoptotic cell death and in agreement with previous findings [40,45]. This arrest correlated with the downregulation of *cyclin D1* and *cyclin E1* gene expression, two critical regulators of G1/S transition [46,47]. In addition, cisplatin triggered a pro-inflammatory response, as shown by the inducing *TNF-α* and *IL-6* gene expression, with no significant effect on the expression of anti-inflammatory cytokine *IL-10* gene. TNF-α may initiate extrinsic apoptosis through binding to its receptor (TNF-R1), recruitment of Fas-associated death domain protein (FADD), and cleavage of caspase-8/-10, which further activates caspase-3 and upregulates *Bax* expression [48]. Additionally, both TNF-α and IL-6 promote immune cell recruitment and phagocytosis of apoptotic cells [49,50].

In parallel, AM in combination with cisplatin exhibited significant synergic anti-tumor properties on the 4T1 breast cancer cell line, primarily through its ability to enhance the effect of cisplatin alone on cell viability, with CI < 1. Therefore, it seemed interesting to investigate the effect of AM on 4T1 cells. The anti-proliferative and anti-adhesive effects of AM were notably more pronounced after 48 h of treatment, with over a 20 and 16-fold reduction compared to untreated cells, suggesting a time-dependent potentiation of its cytotoxic action. Flow cytometric analysis using AV/PI staining confirmed that AM induces apoptotic cell death at a concentration of 1500 µg/mL after 24 and 48 h of exposure. However, no significant changes in the ΔΨm or in caspase-3/7 activity were observed. These findings were supported by RT-qPCR and Western blot data, which showed a downregulation of *caspase-3* gene expression without activation of the corresponding protein or of upstream caspases, i.e., caspase-9, caspase-7. Altogether, the data suggest that AM triggers apoptosis through unconventional mechanisms bypassing the intrinsic mitochondrial pathway and involving alternative modes of cell death [51]. Notably, AM appeared to trigger autophagic cell death by modulating key molecular regulators of the autophagy pathway. AM significantly inhibited *mTOR* gene expression facilitating activation of the ULK1 complex and subsequent initiation of phagophore formation [52]. This was accompanied by upregulation of *Beclin1* gene, a critical autophagy initiator. Depending on its interactions, Beclin1 may either contribute to autophagosome formation or, when complexed with activated caspase-8, promote apoptotic signaling via Bcl-2 inhibition [53]. The observed increase in *LC3* gene expression and LC3-I to LC3-II conversion at 48 h further support the induction of complete autophagy including autophagosome maturation and subsequent fusion with lysosomes.

At the intracellular level, AM modulated the PI3K/Akt/mTOR signaling pathway. After 24 h of treatment, a marked increase in Akt protein cleavage was observed indicating a transient pathway activation. This effect was partially reduced at 48 h, suggesting a rapid turnover of the pathway possibly through autophagy-mediated degradation of Akt or turnover regulatory mechanisms. As a consequence of this early Akt activation, the expression of pro-inflammatory cytokine gene *TNF-α* was significantly upregulated.

Interestingly, the anti-inflammatory cytokine gene expression *IL-10* was also induced, indicating a dual immunomodulatory role of AM within the tumor microenvironment [54]. This complex cytokine profile suggests that AM may contribute to shaping both the inflammatory response and immune-mediated tumor clearance [55]. Moreover, AM treatment significantly reduced the tumorigenic potential of 4T1 cells, as reflected by the complete absence of colony formation after 48 h at the highest tested concentration (1500 µg/mL). A similar effect was observed in carcinoma cell lines (A431, UW-BCC1) after 48 h of incubation [56].

In parallel, cell cycle analysis revealed a notable accumulation of cells in the Sub-G1 phase at 24 h, indicative of apoptotic DNA fragmentation. After 48 h, cells accumulated in both Sub-G1 and S phases suggesting the induction of DNA damage, replication stress and a blockage in cell cycle progression [57]. At the molecular level, AM selectively downregulated *cyclin E1* gene expression, a key regulator of the G1/S transition, without significantly altering *cyclin D1* mRNA levels. This indicates a specific arrest at the late G1 or early S phase, interfering with DNA synthesis and cell cycle progression. These results are in line with previous reports describing Sub-G1 arrest and cell cycle disruption following AM treatment in various cancer models [58,59,60], thereby reinforcing the potential of this plant extract as a multi-targeted anti-cancer agent.

According to recent scientific reports, approximately 23% of cancer patients use medicinal plants, among whom 27% are women diagnosed with breast cancer [61]. Although phytotherapy is increasingly recognized as a complementary strategy in oncology, it cannot substitute for standard-of-care treatments such as chemotherapy. Nonetheless, certain plant-derived compounds have demonstrated selective cytotoxicity toward tumor cells, sparing normal cells at low doses. In this context, hydro-alcoholic extracts of the AM leaf have been shown to exert cytotoxic effects on cancer cells while preserving the viability of normal cells [62]. In light of these properties, we evaluated the combination of AM with cisplatin, aiming to enhance anti-tumor efficacy and modulate chemotherapy-associated toxicities. Our results demonstrate that AM and cisplatin combination exhibits more cytotoxic effects on triple negative 4T1 murine breast cancer cells compared to either treatment alone. Notably, this combination appeared to shift the cisplatin-induced cell death mechanism from classical intrinsic apoptosis toward autophagy suggesting engagement of an alternative death pathway. Additionally, AM restored mitochondrial membrane potential disrupted by cisplatin, indicating a protective effect on mitochondrial function that may contribute to improved tumor cell elimination with reduced cytotoxic burden. Taken together with previous data, these findings support the potential of AM as a valuable adjuvant to chemotherapy [63,64]. At the molecular level, AM prevented *caspase-3* gene expression and, in combination with cisplatin inhibited both expression and cleavage of caspase-3 and -7 proteins after 48 h. This combination also reduced activation of the PI3K/Akt/mTOR signaling pathway, particularly through inhibition of Akt phosphorylation and *mTOR* gene expression. These alterations in survival signaling may underlie the observed shift in the mode of cell death. Furthermore, the combination significantly enhanced *Bax* gene expression and suppressed *Bcl-2* gene expression, promoting mitochondrial outer membrane permeabilization and facilitating autophagic responses. Upregulation of *Beclin1* confirmed the initiation of autophagy which was further supported by increased *LC3* mRNA levels and the conversion of LC3-I to LC3-II, indicating active autophagosome formation and maturation. Importantly, the AM–cisplatin combination also displayed a notable anti-inflammatory effect, counteracting the pro-inflammatory cytokine surge typically associated with cisplatin. Specifically, *TNF-α* gene expression was downregulated, while expression of the anti-inflammatory cytokine *IL-10* gene was enhanced. These findings are consistent with previous in vivo studies, such as those by Silihe et al. (2023), who demonstrated that AM leaf and fruit extracts reduced TNF-α and IL-6 serum levels in DMBA-induced breast cancer models [65]. Similarly, anti-inflammatory effects have been reported in diabetic rats [66] and in LPS-stimulated RAW264.7 macrophages, where AM reduced secretion of inflammatory mediators [67]. AM may influence cisplatin sensitivity by modulating pathways involved in apoptosis, autophagy, and inflammation. Although the role of MAST1 in cisplatin resistance has been well established, its modulation by AM remains to be investigated and represents an important direction for future studies to better understand its potential in overcoming chemoresistance [68]. The dual therapy AM–cisplatin exhibited a synergistic effect on tumorigenic potential, significantly reducing colony formation in a concentration- and time-dependent manner, with complete inhibition observed at 500 and 1500 µg/mL after 48 h. Finally, the combination reinforced cisplatin-induced cell cycle arrest, as evidenced by Sub-G1 accumulation after 48 h. This effect was associated with the downregulation of *cyclin D1* gene expression as early as 12 h, indicating interruption of G1-phase progression and commitment to cell death. Altogether, these findings highlight the potential of AM to enhance the therapeutic efficacy of cisplatin while reducing its deleterious effects particularly through modulation of mitochondrial integrity, autophagy, inflammatory responses, and cell cycle regulation.

Given that cisplatin is commonly associated with chemoresistance and adverse side effects, its combination with AM could offer a novel therapeutic approach aimed at reducing cisplatin doses, improving tumor cell chemosensitivity, and limiting toxicity toward normal cells. These results support the potential of plant-based adjuvants in combination chemotherapy regimens for breast cancer.

## 5. Conclusions

In conclusion, our results highlight the therapeutic potential of AM as a complementary agent to minimize cisplatin toxicity in the treatment of breast cancer. In addition to its intrinsic cytotoxic effects on tumor cells, AM demonstrated the ability to restore mitochondrial integrity, induce autophagy-mediated cell death, modulate pro- and anti-inflammatory cytokines expression and interfere with key oncogenic pathways such as PI3K/Akt/mTOR. Interestingly, these multi-targeted actions not only enhance the anti-tumor efficacy of cisplatin but may also contribute to reducing its associated toxicity. Given these promising results, AM represents a strong candidate for integrative oncology approaches and could be used as complementary medicine to standard therapies for breast cancer treatment. Future studies should aim to validate these findings in vivo, assess pharmacokinetic interactions, and explore the safety and efficacy of this combination in clinical settings.

## Figures and Tables

**Table 1:** Antibodies used for Western blot experiment.

Antibody/Catalog Number	Supplier	Molecular Weight	Host	Dilution
Caspase3 9662S	Cell Signalling Technology (Danvers, MA, USA)	Pro 35 KDa	Rabbit	1/1000
		Cleaved 17 KDa		
Caspase7 9492S	Cell Signalling Technology	Pro 35 KDa	Rabbit	1/1000
		Cleaved 20 KDa		
Caspase9 9508S	Cell Signalling Technology	Pro 39 KDa	Mouse	1/1000
		Cleaved 37 KDa		
PARP 46D11	Cell Signalling Technology	Pro 116 KDa	Rabbit	1/1000
		Cleaved 89 KDa		
Akt 9272S	Cell Signalling Technology	60 KDa	Rabbit	1/1000
Phospho-Akt (Thr 308) 2965S	Cell Signalling Technology	60 KDa	Rabbit	1/1000
Anti-Rabbit IgG, HRP-Linked Antibody 7074S	Cell Signalling Technology	-	-	1/1000
Actin A2228	Sigma Aldrich (St. Louis, MO, USA)	42 KDa	Mouse	1/10,000
LC3 L8918	Sigma Aldrich	LC3-I 18 KDa	Rabbit	1/1000
		LC3-II 16 KDa		
Anti-Mouse (IgG2a) HSP70 B0703	Santa Cruz Biotechnology (Dallas, TX, USA)	-	-	1/1000

**Table 2:** Primers used for RT-qPCR experiment.

Gene and Accession Number	Forward Primers	Reverse Primers
*GADPH* NM_008084.3	5′-TGAGGACCAGGTTGTCTCCT-3′	5′-CCCTGTTGCTGTAGCCGTAT-3′
*Caspase3* NM_009810	5′-GAGGCTGACTTCCTGTATGCTT-3′	5′-AACCACGACCCGTCCTTT-3′
*BAX* NM_007527	5′-GTGAGCGGCTGCTTGTCT-3′	5′-GGTCCCGAAGTAGGAGAGGA-3′
*Bcl2* NM_009741	5′-GTACCTGAACCGGCATCTG-3′	5′-GGGGCCATATAGTTCCACAA-3′
*Beclin1* NM_001290692.1	5′-GATGAGGCACTGAGGGCTAC-3′	5′-TAAGAGGGAGAGGGGGCATC-3′
*mTOR* NM_020009.2	5′-AGTCCAAGTCAAGTC-3′	5′-AGAGAGGGATTGATCTCGCAAGA-3′
*LC3* NM_025735.3	5′-TGTCCTGGATAAGACCAAGTTTCTG-3′	5′-ACCATGCTGTGCTGGTTGAC-3′
*HIF1α* NM_001422143.1	5′-GTGCACCCTAACAAGCCGGGG-3′	5′-CCGTGCAGTGAAGCACCTTCCA-3′
*TNFα* NM_013693.2	5′-CACCGTCAGCCGATTTGC-3′	5′-TGAGTTGGTCCCCCTTCTCC-3′
*IL-6* NM_031168.1	5′-GAAACC GCTATGAAGTTCCTCTCTG-3′	5′-TGTTGGGAGTGGTATCCTCTGTGA-3′
*IL-10* NM_010548.2	5′-AAGGCAGTGGAGCAGGTGAA-3′	5′-CCAGCAGACTCAATACACAC-3′
*Cycline D1* NM_001379248.1	5′-GCCCGAGGAGCTGCTGCAAA-3′	5′-GCCTTGCATCGCAGCCACCA-3′
*Cycline E1* NM_007633.2	5′-CTGAGAGATGAGCACTTTCTGC-3′	5′-GAGCTTATAGACTTCGCACACCT-3′

**Table 3:** Determination of the interaction between *Annona muricata* leaf extract and CDDP using Chou and Talalay method on 4T1 cell viability.

Dose AM (µg/mL)	Dose CDDP (µM)	DRI AM	DRI CDDP	Fa	CI
50	1	1605.69	20.805	0.21	0.049
500	5	5180.15	15.954	0.127	0.063
1500	10	16.691	1.291	0.36	0.835

## Data Availability

The original contributions presented in this study are included in the article. Further inquiries can be directed to the corresponding author.

## Abbreviation

DNA	Deoxyribonucleic acid
ROS	Reactive oxygen species
AM	*Annona muricata*
Cis	Cisplatin
PI	Propidium iodide
ΔΨm	Transmembrane mitochondrial potential
PBS	Phosphate Buffered Saline
IC_50_	Inhibitory concentration 50%
PARP	Poly (ADP-ribose) polymerase
RT-qPCR	Quantitative reverse transcription polymerase chain reaction
IL-6	Interleukin-6
IL-10	Interleukin-10
TNF-α	Tumor necrosis factor-alpha
WHO	World Health Organization
HIF1-α	Hypoxia-inducible factor1-alpha
ULK1	Unc-51-like kinase 1
PI3K	Phosphoinositide 3-kinase
mTOR	Mammalian Target of Rapamycin
FBS	Fetal bovine serum
FDA	Fluorescein diacetate
AV	Annexin V
Bax	Bcl-2 associated X
CDDP	Cis-diamminedichloroplatinum(II)/cisplatin
